# Dynamics of the Bacterial Intermediate Filament Crescentin *In Vitro* and *In Vivo*

**DOI:** 10.1371/journal.pone.0008855

**Published:** 2010-01-25

**Authors:** Osigwe Esue, Laura Rupprecht, Sean X. Sun, Denis Wirtz

**Affiliations:** 1 Department of Pharmaceutical Development, Genentech, South San Francisco, California, United States of America; 2 Department of Chemical and Biomolecular Engineering and Physical Science Oncology Center, The Johns Hopkins University, Baltimore, Maryland, United States of America; 3 Department of Biomedical Engineering, Boston University, Boston, Massachusetts, United States of America; 4 Department of Mechanical Engineering, The Johns Hopkins University, Baltimore, Maryland, United States of America; Dartmouth College, United States of America

## Abstract

**Background:**

Crescentin, the recently discovered bacterial intermediate filament protein, organizes into an extended filamentous structure that spans the length of the bacterium *Caulobacter crescentus* and plays a critical role in defining its curvature. The mechanism by which crescentin mediates cell curvature and whether crescentin filamentous structures are dynamic and/or polar are not fully understood.

**Methodology/Principal Findings:**

Using light microscopy, electron microscopy and quantitative rheology, we investigated the mechanics and dynamics of crescentin structures. Live-cell microscopy reveals that crescentin forms structures *in vivo* that undergo slow remodeling. The exchange of subunits between these structures and a pool of unassembled subunits is slow during the life cycle of the cell however; *in vitro* assembly and gelation of *C. crescentus* crescentin structures are rapid. Moreover, crescentin forms filamentous structures that are elastic, solid-like, and, like other intermediate filaments, can recover a significant portion of their network elasticity after shear. The assembly efficiency of crescentin is largely unaffected by monovalent cations (K^+^, Na^+^), but is enhanced by divalent cations (Mg^2+^, Ca^2+^), suggesting that the assembly kinetics and micromechanics of crescentin depend on the valence of the ions present in solution.

**Conclusions/Significance:**

These results indicate that crescentin forms filamentous structures that are elastic, labile, and stiff, and that their low dissociation rate from established structures controls the slow remodeling of crescentin in *C. crescentus*.

## Introduction

The discovery of an intermediate filament (IF)-like protein in the bacterium *Caulobacter crescentus*, crescentin [1], [2], suggests that all three major types of cytoskeletal filamentous proteins are represented in the prokaryotic world: actin homologs (MreB/Mbl/ParM) [3]–[5], a tubulin homolog (FtsZ) [6]–[9], and an IF homolog (FilP/Crescentin) [10], [11]. The amino acid sequence of crescentin shares 25% identity and 40% similarity with that of IF cytoplasmic protein keratin 19, and 24% identity and 40% similarity with that of IF nuclear protein lamin A. This sequence similarity stems mainly from a regular 7-residues repetitive pattern of alternative hydrophobic and hydrophilic residues [1], [10].

Similar to FilP in Streptomyces [10], crescentin plays a critical shape-determining function in wild-type crescent-shaped *C. crescentus* [12], [13]. In young cultures of *C. crescentus*, crescentin forms a continuous filamentous structure along the inner concave side of the cells [1], [11]. In old stationary phase cultures, *C. crescentus* cells become helical and crescentin organizes into a helical structure. *C. crescentus* cells lacking crescentin lose their curvature and become straight-rod shaped. The mechanism by which crescentin causes cell curvature is not fully understood however studies suggest that when crescentin interacts with the cell wall during growth, it creates an elongation rate differential that contributes to cell curvature [2], [12]. Another possible mechanism of curvature could be explained by the mislocalization of crescentin filaments following the disruption of peptidoglycan synthesis [1]. Whether crescentin forms structures that are dynamic or polar are largely unanswered questions. *In vitro*, crescentin assembles into filaments of morphology and diameter similar to those formed by eukaryotic IFs [1], including vimentin and keratin. However, little is known about the mode and kinetics of crescentin filament assembly in vitro and whether crescentin structures feature the mechanical properties required for affecting bacterial cell mechanics.

Despite the important mechanical function that IFs have to perform, they form structures that are relatively dynamic [14]–[17], albeit much less dynamic than actin and microtubule structures [18]–[25]. For example, IF vimentin-GFP in fibroblasts forms networks that continuously change their organization. Fluorescence recovery after photobleaching (FRAP) studies show that vimentin-GFP rapidly recovers its fluorescence with a half lifetime of 5–14 min [15]. Keratin-GFP also recovers its fluorescence, but with a much longer halftime of ∼100 min [14]. It is unknown whether crescentin, like its eukaryotic counterparts, forms dynamic structures that would exchange subunits with a pool of unpolymerized crescentin *in vivo*.

Here we present a biochemical and biophysical characterization of the dynamics of crescentin *in vitro* and *in vivo*. Electron microscopy (EM) and quantitative rheology *in vitro* are combined with FRAP, fluorescence loss in photobleaching (FLIP), and kinetics modeling *in vivo* to investigate the assembly, disassembly, organization, dynamics, and micromechanics of crescentin filaments. These studies indicate that, *in vivo*, crescentin forms filamentous structures that are not static, but undergo slow remodeling dynamics. Similar to eukaryotic IFs, crescentin filaments form structures that are both viscous and elastic, i.e. structures that are stiff at short time scales and flow at long time scales. However, unlike most eukaryotic IFs, crescentin filaments display no strain-induced hardening and poor mechanical resilience. Together these results indicate that crescentin forms stiff, yet dynamic, structures and that its dissociation rate, not the assembly rate, controls the rate of remodeling of crescentin structures in *C. crescentus*.

## Materials and Methods

### Purification of Crescentin

*E. coli* BL21 (DE3) cells, described in [1], transformed with the pET 28a(+) vector derived construct containing the C. crescentus crescentin encoding gene [1], were inoculated at 37°C in 2× YT media supplemented with 100 µg/ml ampicillin until the culture reached an OD_600_ of 0.6. Production of polyhistidine-tagged crescentin was induced with 1 mM isopropyl-beta-D-thiogalactopyranoside (IPTG) for 3 h. The cells were spun down at 6,000×g for 15 min, quickly frozen by liquid nitrogen, and stored at -20°C for further use. Cell pellets were thawed and resuspended in lysis/wash buffer (50 mM Tris buffer, pH 8.0, 300 mM NaCl, 1 mM PMSF, 6 M urea) containing lysozyme. The mixture was sonicated and DNase I was added. The mixture was centrifuged (150,000×g) for 1 h and the supernatant was subjected to affinity chromatography using the Talon metal affinity resin (BD Biosciences Clontech, CA) under denaturing conditions (6 M Urea). To remove the polyhistidine tag, biotinylated thrombin was added to fractions containing crescentin after dialysis in a stepwise manner into thrombin-cleavage buffer (20 mM Tris buffer, pH 8.4, 1 M urea, 100 mM NaCl, 2.5 mM CaCl_2_). The cleaved poly-histidine tag bound to biotinylated thrombin was removed with a streptavidin resin (Novagen). Crescentin was then further purified by gel filtration using the sephacryl S-300 resin (Sigma) equilibrated in a buffer containing 5 mM Tris buffer, pH 8.4, 1 mM EDTA, and 1 M urea. Before use, the purified protein was dialyzed in storage buffer (5 mM Tris-HCl, pH 8.4) at 4°C and centrifuged (150,000×g) for 1 h.

### Confocal Microscopy, FLIP and FRAP

*C. crescentus* cells described in [1] were immobilized on 1% agarose pads and visualized in PYE media at room temperature using a Zeiss LSM 510 confocal microscope fitted with a DIC 100× objective. Images of crescentin-GFP were obtained by excitation at 488 nm and emission at 545 nm. Photobleaching was performed according to Yoon *et al*. [14] by exposing a 100% intensity of 488 nm laser to a region of interest. Fluorescent images were acquired before and after photobleaching using a custom time-lapse program. We note that because of the small size of bacterial cells, a large fraction of the cell volume is always photobleached. Therefore, full recovery of FRAP cannot occur and an estimation of immobile fractions is impossible. Nevertheless, the rate of recovery can be relatively reliably estimated. In FLIP experiments, a region of the cell was repeatedly bleached and imaged with the same image acquisition software as FRAP. The FLIP and FRAP intensities were corrected for the total decrease in fluorescence due to photobleaching. Mean FRAP and FLIP profiles were analyzed by simple kinetic modeling, as described in Daniels et al, [26].

### *In Vitro* Filament Assembly

Crescentin was assembled at room temperature by adding 1 volume of polymerizing buffer (500 mM MES buffer, pH 6.5) to 9 volumes of crescentin in storage buffer. The polymerizing buffer was supplemented with 1.6 M NaCl, 1.6 M KCl, 50 mM CaCl_2_, or 50 mM MgCl_2_, as needed. Protein concentration was measured using the calibrated BCA (bicinchoninic acid) assay.

### Electron Microscopy

The ultrastructure of crescentin filaments was examined by electron microscopy. Crescentin was incubated in assembly buffer (50 mM MES buffer, pH 6.5) obtained by mixing 1 volume of polymerizing buffer to 9 volumes of storage buffer. Ten µl of solution was placed on each collodion-coated electron microscopic grid. Grids were washed with assembly buffer and stained with 2% uranyl acetate solution [27]. Electron microscopy was performed at the Imaging Center in Yale University with a Philips 410 transmission electron microscope at magnifications between 50,000× and 105,000×, as indicated.

### Quantitative Rheology

Quantitative rheology was used to measure the mechanical properties of crescentin during filament assembly and at steady state. After addition of the polymerization buffer (loading deadtime, 30 s), crescentin suspensions were placed between the 50-mm diameter cone (cone angle = 4°) and plate of a strain-controlled rheometer (ARES-100 TA Instrument, Piscataway, NJ) [28]. The plate is coupled to a computer-controlled motor, which applies either steady or oscillatory shear deformations of controlled frequency and amplitude. The cone is connected to a torque transducer, which measures the stress induced in the crescentin solutions by the applied shear deformations. First, oscillatory shear deformations of a small amplitude of 1% and a frequency of 1 rad/s were applied every 30 s and the resulting oscillatory stress was recorded until it reached a steady state value. Here we report the in-phase and out-of-phase components of the stress divided by the amplitude of the deformation (1%), i.e. the elastic modulus, G′, and viscous modulus, G″, respectively, as well as the phase angle, δ = tan^−1^(G″/G′). Next, oscillatory deformations of small amplitude (1%) and frequency between 0.01 and 100 rad/s were applied to measure the frequency-dependent elastic modulus, G′(ω), and viscous modulus, G″(ω), of the crescentin filament networks. Finally, deformations of fixed frequency (1 rad/s) and deformation amplitude between 0.1% and 1000% were applied to measure the elastic and viscous moduli as a function of deformation amplitude γ, G′(γ) and G″(γ), respectively.

## Results

### *In Vivo* Dynamics of Crescentin-GFP

**Figure 1:**
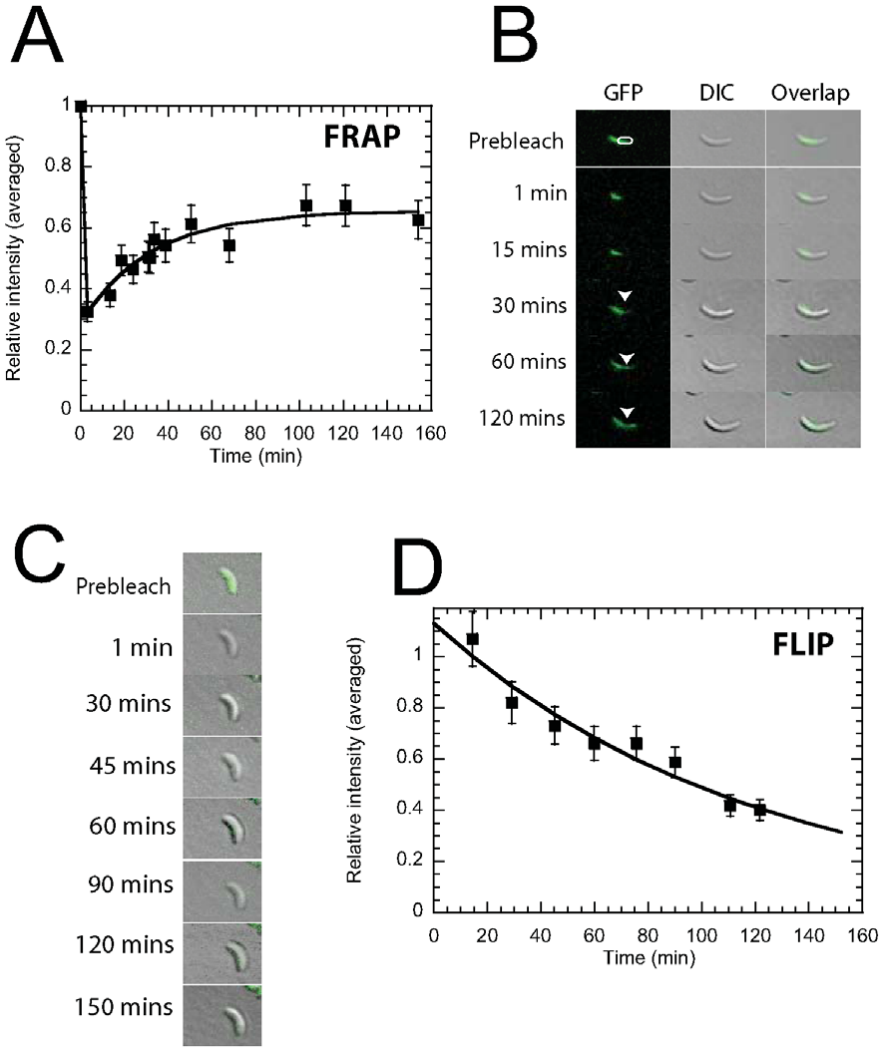
*In vivo* dynamics and FRAP/FLIP analysis of crescentin-GFP in *C. crescentus*. ***A***, crescentin-GFP FRAP kinetics. The half-life time for recovery is t_1/2_ = 26±1.9 min (n = 12). The curve is a fit based on a FRAP kinetics model. ***B***, Time-lapsed fluorescence microscopy images of crescentin-GFP following photobleaching in the indicated region of the cell. Columns show selected images before and after photobleaching. Arrow heads show fluorescence recovery in bleached region (white circle) of cells. Columns correspond to DIC, GFP fluorescence and overlay, respectively. ***C***, Overlaid DIC and crescentin-GFP fluorescence micrographs in a cell that has been completely photobleached. The cell does not recover its fluorescence. ***D*** **,** Crescentin-GFP FLIP kinetics. A region in the cell was repeatedly photobleached for 120 min and the loss in fluorescence in the rest of the cell was measured. The rate of FLIP is 0.50±0.18 h^−1^ (*n* = 12 cells) (mean ± standard error). The curve is a fit based on a FLIP kinetics model.

We used FRAP to investigate the dynamics of crescentin-GFP *in vivo*. A defined region of individual *C. crescentus* cells was bleached with a laser pulse of 500 ms. The kinetics of recovery, reflecting the mobility of crescentin-GFP, was monitored using time-lapsed fluorescence confocal microscopy. After photobleaching, the fluorescence intensity recovered slowly (Fig. 1, A and B). The mean half time of recovery was 26±2 min (mean ± standard error; *n* = 12 cells). This time represents a significant fraction of the life cycle time of a *C. crescentus* cell grown on agarose pads (∼150 min). The mean FRAP intensity profile was well fitted by an exponential curve obtained from a simple kinetics model of exchange of crescentin-GFP subunits between bleached and unbleached regions of the cell [29] (Fig. 1A). Partial photobleaching did not appear to affect cell growth over 2 h (e.g. Fig. 1, A and B). On the other hand, cells that were completely bleached appeared to stop growing and did not show any significant fluorescent recovery (Fig. 1C). We found no polarity in crescentin structures, which would be indicated by a net migration of the bleached region. This result indicates that if crescentin filaments are indeed polar, then they are arranged in an anti-polar fashion *in vivo*.

FRAP measurements were complemented by FLIP studies. FLIP is a method that is particularly well suited to probe the dissociation dynamics of GFP-labeled proteins from established structures. A defined crescentin-GFP-containing region of the cell was repeatedly bleached using a 500-ms laser pulse and the cell was imaged by time-lapsed confocal microscopy. The fluorescence intensity in the unbleached region of the cell declined exponentially, with a rate of 0.50±0.18 h^−1^ (*n* = 12 cells) (Fig. 1D). This slow rate of FLIP is consistent with the slow rate of recovery in the FRAP experiments. Together the results of these FRAP and FLIP studies suggest that the slow dynamics of crescentin-GFP is controlled by the slow dissociation of crescentin subunits from established crescentin structures. We used time-lapsed confocal fluorescence and DIC microscopy to investigate the organization of crescentin-GFP in growing *C. crescentus* cells. Crescentin structures formed structures that spanned the length of *C. crescentus* cells over most of their life cycle. Significant rearrangement of the crescentin-GFP structures occurred only at the onset of cell division, just before daughter cell separation, at the site of division. But FRAP analysis of crescentin-GFP indicated that crescentin dynamics were also slow in a dividing cell, with a half time of recovery of t_1/2_ = 27±3 min (not shown). Together, results from FRAP, FLIP, and time-lapsed microscopy experiments indicate that crescentin structures *in vivo* undergo slow remodeling and the exchange of subunits between these structures and a pool of unassembled subunits is very slow during the life cycle of the cell.

### Crescentin Forms Elastic Structures

IFs (including vimentin, keratins, lamins, etc.) present distinct mechanical features that are significantly different from the two other major cytoskeleton filamentous proteins, F-actin and microtubule. Specifically, eukaryotic IFs form structures that are elastic, recover their elasticity after shear, and increase their stiffness under mechanical stress, even in the absence of auxiliary proteins [30], [31]. We used quantitative rheology to determine whether the IF-like bacterial protein crescentin shared these mechanical properties and, therefore, test whether crescentin could be mechanically classified as an IF. The mechanical properties of crescentin were probed using a cone-and-plate rheometer, which monitored both the elastic modulus, G′ (which measures the propensity of the network to rebound after shear), and the viscous modulus, G″ (which measures how much the specimen can flow).

**Figure 2:**
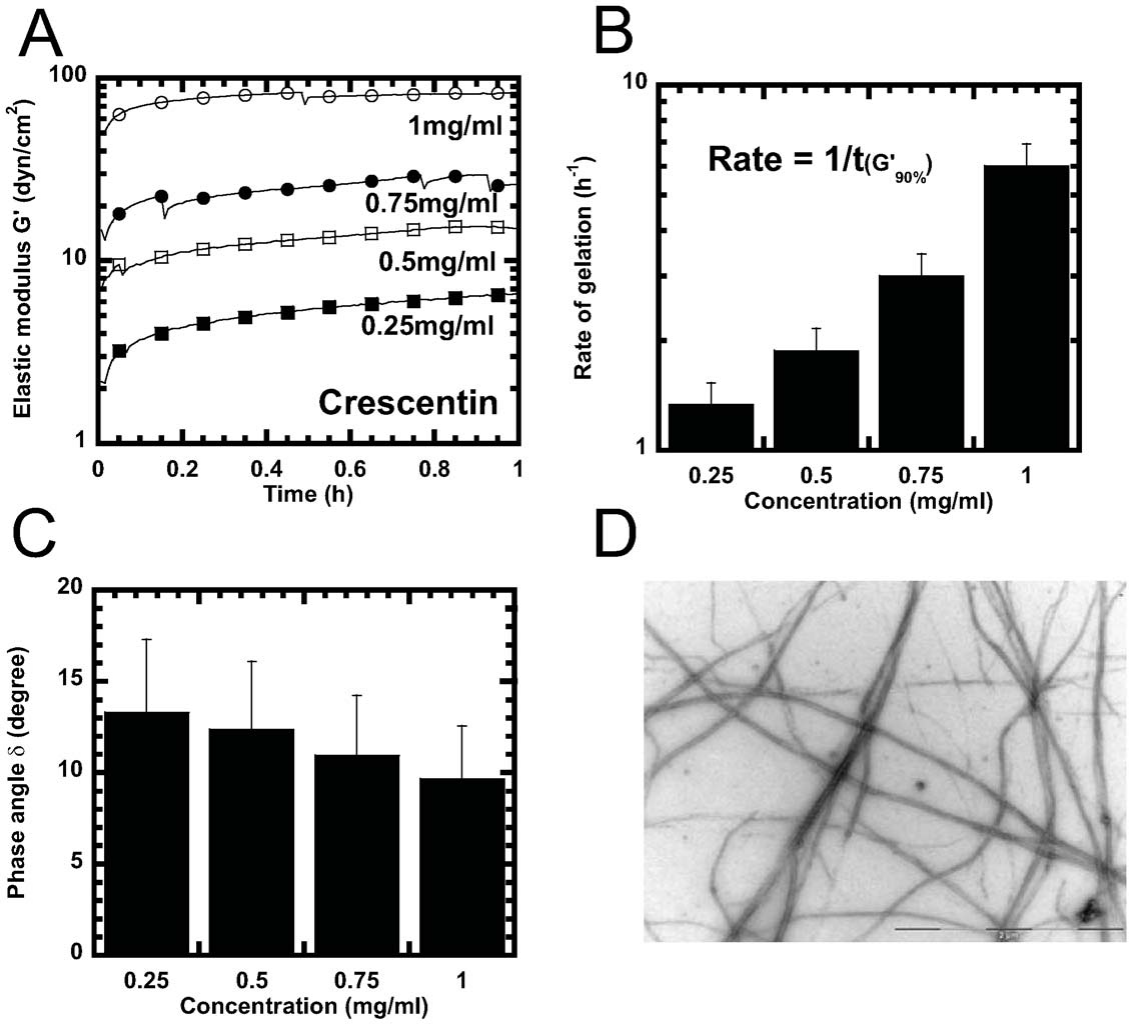
Gelation kinetics and steady-state mechanical properties of crescentin. ***A***, Time-dependent elastic modulus, G′, after the onset of crescentin assembly (dead time for specimen loading in the rheometer was 30 s). Crescentin concentrations: 0.25 mg/ml (filled squares), 0.5 mg/ml (open squares), 0.75 mg/ml (filled circles), and 1 mg/ml (open circles). ***B***, Rate of gelation as a function of protein concentration measured as the inverse of the time required for the network elasticity to reach 90% of its plateau value. ***C***, phase angle, δ, of crescentin structures as a function of concentration. A phase angle of 90° describes the rheological behavior of a liquid (e.g. glycerol); a phase angle of 0° describes the rheological behavior of an elastic solid (e.g. a stiff rubber). Phase angle was evaluated at a frequency of 1 rad/s and a strain amplitude of 1%. ***D***, Crescentin filaments (0.2 mg/ml) visualized by negative staining and EM. Bar, 2 µm

As expected, crescentin in storage buffer displayed no elasticity and had a viscosity comparable to that of buffer. Upon addition of polymerizing buffer, crescentin progressively displayed elasticity which was much higher than that of crescentin in storage buffer or polymerizing buffer without proteins (Fig. 2A). The elasticity of crescentin reached a plateau value, which was significantly higher than that of other eukaryotic IFs (Table 1). The rate of gelation of crescentin filament networks, calculated as the inverse of the time it takes to reach 90% of the steady state plateau value, increased steadily with crescentin concentration (Fig. 2B). The phase angle of crescentin filament network, δ = tan^−1^(G″/G′)∼10°, which compares the relative magnitudes of viscous and elastic moduli, was lower than that of both F-actin and microtubule network. However, the phase angle was comparable to that of networks of eukaryotic IF's (Table 1 and Fig. 2C), which indicates that crescentin filaments flowed under mechanical stress similarly to eukaryotic IFs. The elasticity of crescentin filament suspensions depended highly on concentration, with a scaling consistent with a power law, G′(c)∼c^3.5^ (Fig. 2A). By comparison, the elasticity of eukaryotic filament networks increased less steeply with protein concentration: G′∼c^1.2^ for F-actin; G′∼c^1.5^ for keratin K5-K14; and G′∼c^1.4^ for lamin B1 (Table 1). Complementary electron microscopy (EM) revealed that crescentin formed extensive filament networks under assembly conditions (Fig. 2D).

**Table 1:** Mechanical properties of crescentin and its eukaryotic counterparts *in vitro*.

Cytoskeleton protein	Elasticity (dyn/cm^2^)	Phase angle (°)	Resilience (%)	Exponent G′(c)∼c^n^	pH	Source
Crescentin	82±10	10±3	2±1	3.5	6.5	This work
Lamin B1	1±1	9±2	200±30	1.4	8.8	Panorchan *et al.* 2004
Vimentin	4±2	9±2	10±3	NA	7.4	Esue *et al.* 2006
Keratin K5-K14	7±2	4±2	200±30	1.5	7.4	Yamada *et al*. 2002
Keratin K8-K18	5±2	5±2	100±20	0.6	7.4	Yamada *et al*. 2003
F-actin	10±3	30±5	5±2	1.2	7.0	Xu et al. 2000
Microtubule	6±2	40±6	2±1	NA	6.8	Unpublished results

**Figure 3:**
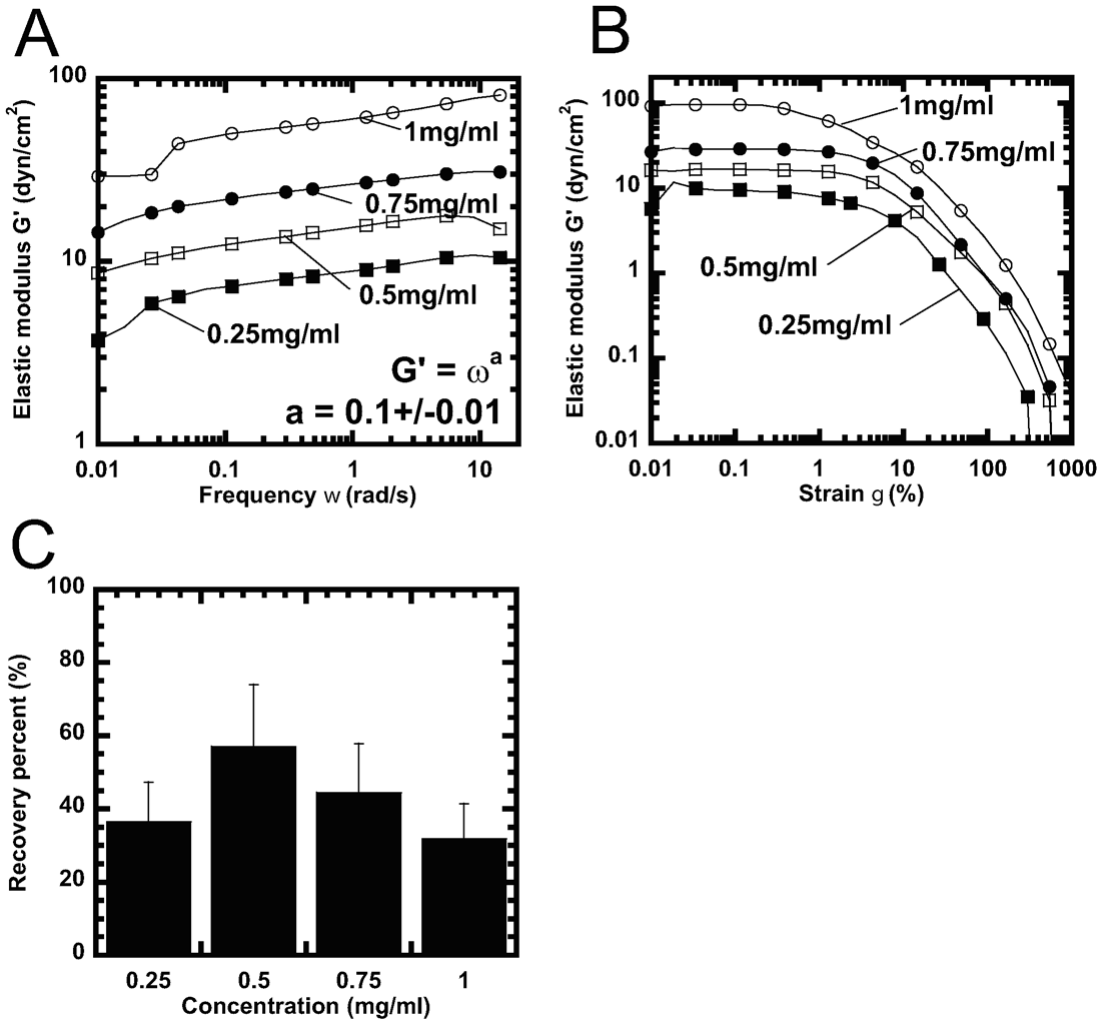
Response of crescentin to mechanical deformation. ***A***, Frequency-dependent elastic modulus, G′(ω), of crescentin as different concentrations. ***B***, Elasticity, G′, of crescentin as a function of strain amplitude, γ (see Methods section). Crescentin does not undergo strain-hardening, whereby G′ would increase with γ. Crescentin concentrations in panels A and B are 0.25 mg/ml (filled squares), 0.5 mg/ml (open squares), 0.75 mg/ml (filled circles), and 1 mg/ml (open circles). ***C***, Recovery of the elasticity of crescentin following the application of a large short-lived shear deformation as a function of crescentin concentration. Percent recovery is defined as the ratio of the recovered elasticity after application of a couple of oscillatory shear deformations of 1000% and the initial elasticity. Elasticity was evaluated at a frequency of 1 rad/s and a strain amplitude of 1%.

Elasticity, G′, and phase angle, δ, were measured using a cone-and-plate strain-controlled rheometer, which applied oscillatory shear deformations of small 1%-amplitude and a frequency of 1 rad/s. Rheological parameters G′ and δ were measured at steady state, i.e. after these parameters had reached a steady state after onset of assembly. The phase angle measures the delay in the response of the stress induced in the filament networks by the rheometer. An elastic solid shows no delay (phase angle of 0°); a viscous liquid without elasticity like glycerol shows a maximum delay (phase angle of 90°). The mechanical resilience of cytoskeleton proteins is defined as the shear amplitude at which the elastic modulus started to fall (e.g. Fig. 3). Protein concentration for measurements of G′, δ, and resilience was 1 mg/ml. For the range of concentrations for the concentration-dependent elasticity, G′(c), see text and references.

### Mechanical Response of Crescentin Filaments to Shear Stresses

Crescentin filament suspensions were subjected to shear deformations of increasing shear rate and fixed small amplitude. This mechanical test determines the magnitude of spontaneous movements of crescentin filaments within their network, which would relax the stress. Filament movements may be impeded by entanglements and/or inter-filament crosslinking interactions, which give rise to network elasticity. Along with its high network elasticity, crescentin filaments formed networks that behaved as visco-elastic solids, which flowed little when subjected to shear and could elastically resist mechanical shear stresses (Fig. 3). The elasticity of crescentin filaments, G′(ω), showed a weak dependence on the rate of shear (the shear frequency ω) (Fig. 3A); this frequency dependence was similar to those of vimentin and keratin K5-K14 [32]–[34]. This result indicates that crescentin filaments were able to move less readily past one another - and therefore relaxed more slowly the stress during cycles of shear. Crescentin filaments relaxed mechanical stresses at the same rate as their eukaryotic counterparts [32].

We tested the propensity of crescentin filaments to resist shear deformations of increasing amplitude. In contrast to eukaryotic IFs (Table 1), crescentin filaments offered little resistance to shear: they softened at a shear amplitude as low as ∼1%, as indicated by a sharp drop of G′(γ) at γ∼1% (Fig. 3B). This level of mechanical resilience is similar to that offered by F-actin without crosslinking proteins and much lower than the resilience of eukaryotic IFs (Table 1). IFs lamin B1 and keratin, as well as crosslinked F-actin, displayed a resilience that was 10–50 fold higher than crescentin (Table 1). Moreover, these cytoskeletal filaments undergo strain-induced hardening (also called strain-stiffening), whereby elasticity increased steadily with increased mechanical shear, before yielding at high shear amplitudes (Table 1). In contrast, crescentin displayed no strain-hardening (Fig. 3B).

Unlike other networks of filamentous proteins, MreB and F-actin, which recover slowly to only a small fraction (<10%) of their initial elasticity after a large shear deformation [35], [36], crescentin filament networks recovered rapidly to between 30 and 60% of their initial elasticity (Fig. 3C). In the recovery assay, crescentin was allowed to gel until steady state elasticity was reached. Each tested crescentin suspension was then subjected to a couple of oscillatory deformations of 1000% amplitude and frequency of 1 rad/s. As a result of this large deformation, the elasticity dropped to undetectable values. But the elasticity increased rapidly and recovered ∼59% of its initial value for a 0.5 mg/ml gel and ∼30% for a 1 mg/ml gel (Fig. 3C). This rapid recovery is similar to that observed with other IFs [37].

### Divalent, but Not Monovalent, Cations Affect Crescentin Network Assembly and Structure

**Figure 4:**
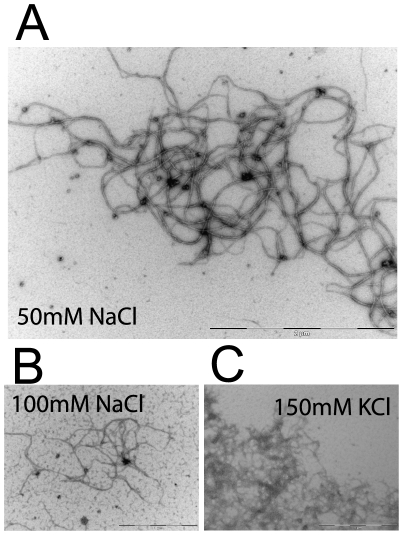
EM of crescentin filaments assembled with monovalent cations. Crescentin filaments (0.2 mg/ml) in the presence of ***A***, 50 mM NaCl (scale bar, 2 µm) ***B***, 100 mM NaCl (scale bar, 1 µm), and ***C***, 150 mM KCl (scale bar, 1 µm) respectively, visualized by negative staining and EM.

**Figure 5:**
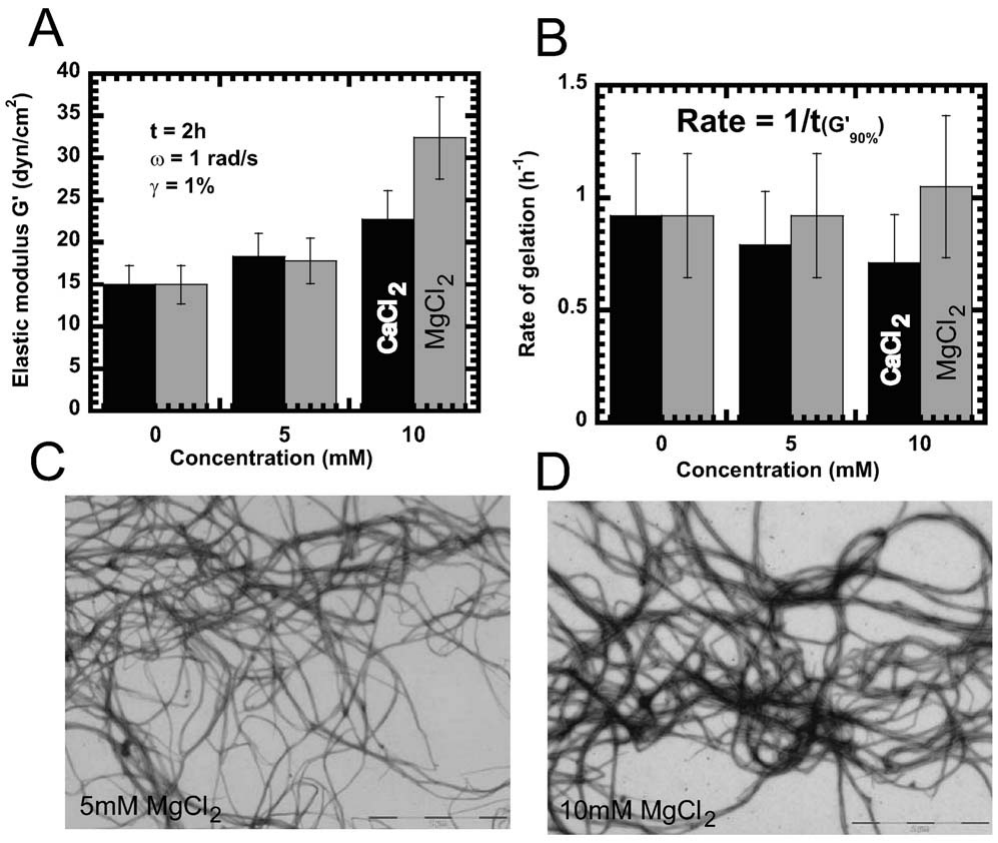
Effect of divalent cations on mechanical properties of Crescentin. ***A***, Steady state elastic modulus, G′, of crescentin structures as a function of MgCl_2_ (grey) and CaCl_2_ (black) concentration. Elasticity was evaluated at a frequency of 1 rad/s and a strain amplitude of 1% after 2 h of gelation. ***B***, Rate of gelation as a function of MgCl_2_ (grey) and CaCl_2_ (black) concentration measured as the inverse of the time required for the network elasticity to reach 90% of its plateau value. Crescentin filaments (0.2 mg/ml) in the presence of ***C***, 5 mM MgCl_2_ (scale bar, 2 µm) and ***D***, 10 mM MgCl_2_ (scale bar, 2 µm), respectively, visualized by negative staining and EM. Symbols correspond to control (closed squares), control with 10 mM CaCl_2_ (open squares), and control with 10 mM MgCl_2_ (closed circles).

The presence of cations affects the kinetics and extent of assembly of most cytoskeletal proteins, including eukaryotic IFs [38]–[40], F-actin, MreB [36], and FtsZ [41]–[43]. Therefore, we studied the effect of monovalent cations (K^+^, Na^+^) and divalent cations (Ca^2+^, Mg^2+^) on crescentin assembly, although these cations are not necessary for crescentin filament assembly (Fig. 2). EM and negative staining showed that the ultrastructure of crescentin filaments was unaffected by low concentrations (≤50 mM) of Na^+^ and K^+^ ions, while the filaments assembled in the presence of Ca^2+^ and Mg^2+^ appeared more bundled and rigid (Fig. 4 and 5). In the presence of 50 mM NaCl, crescentin formed networks composed of highly entangled filaments (Fig. 4A), while in 100 mM NaCl, the extent of filamentous formation was significantly reduced with fragile-looking filaments (Fig. 4B). This trend was similar with K^+^ ions and as shown by EM, crescentin formed a mixture of amorphous aggregates and ordered filaments in the presence of 150 mM KCl (Fig. 4C). Rheology studies complemented these findings to show that 50 mM of either NaCl or KCl did not affect crescentin's assembly, steady state mechanical properties or ability to recover after shear deformation (not shown).

Addition of divalent ions, Ca^2+^ or Mg^2+^, on the other hand made crescentin networks stiffer and more solid-like than networks without cations as indicated by an increase in elastic modulus with increase in divalent ion concentration (Fig. 5A). Crescentin network elasticity increased from ∼15 dyn/cm^2^ ( = 1.5 Pascal), without divalent ions to ∼18 dyn/cm^2^ in the presence of either 5 mM Ca^2+^ or Mg^2+^ and to 22 dyn/cm^2^ and 32 dyn/cm^2^ in 10 mM Ca^2+^ and 10 mM Mg^2+^, respectively (Fig. 5A). The rate of gelation decreased slightly in the presence of Ca^2+^ but increased in the presence of Mg^2+^ (Fig. 5B). This increase in elasticity was also evident from electron micrographs, which revealed that crescentin formed more dense networks consisting of filament bundles in the presence of divalent ions (Fig. 5, C and D). Surprisingly, this increase in filament bundling and network density as assessed by EM did not significantly increase the steady state mechanical properties of crescentin networks.

Similar to other IFs, crescentin networks showed a weak dependence on the frequency of the network elasticity, G′(ω), that was not affected by adding divalent ions during assembly. Together these results indicate that crescentin displays mechanical properties that are unique among cytoskeleton filamentous proteins.

## Discussion

Until recently it was assumed that bacterial cell shape was primarily established and maintained by the glycopeptide polymer peptidoglycan of the cell wall. The degradation of peptidoglycan by lysozyme causes rod-shaped *E. coli* cells to become spherical and mutations of proteins involved in peptidoglycan metabolism affect the shape of various bacteria. However, the recent finding that the cytoplasmic proteins MreB [3], [44], [45] and crescentin [1], [12], which both assemble into filamentous structures *in vitro*, play a critical shape-determining role has changed this simple view of cell morphogenesis in prokaryotes [2], [46], [47].

This paper studies the *in vivo* dynamics and *in vitro* assembly and mechanical properties of the recently discovered crescentin IF-like protein. Crescentin in *C. crescentus* forms structures that are not static, but undergo slow remodeling and allow for little exchange between these structures and a pool of unassembled subunits *in vivo*. Since protein synthesis was not blocked, newly formed crescentin proteins may also contribute to the unassembled subunits *in vivo* as shown by Charbon et al [2]. Moreover, the organization and dynamics of crescentin do not significantly change during the *C. crescentus* cell life cycle. *In vitro*, crescentin shares many structural features and assembly properties with eukaryotic IFs. In particular, crescentin's fast filament assembly leads to the rapid formation of viscoelastic filament networks in a manner similar to its eukaryotic IF counterparts, although crescentin filament assemblies are significantly less mechanically resilient than those formed by eukaryotic IFs.

### Crescentin Dynamics

Crescentin spans the length of *C. crescentus* cells and is mostly stable during the duration of the cell cycle, during which the cell maintains a distinct crescent shape. The time required for recovery of GFP-crescentin in FRAP experiments (t_1/2_ = 26–50 min) [2] represents a substantial fraction of the *C. crescentus* cycle lifetime and is significantly slower than that of other major prokaryotic cytoskeletal proteins, *E. coli* FtsZ (t_1/2_ = 9 s) [48] and *B. subtilis* Mbl (t_1/2_ = 7–10 min) [49]. It is also slower than vimentin dynamics in interphase cells (t_1/2_ = 5–14 min), but faster than the dynamics of cytoplasmic keratins (∼100 min), albeit those times represent small fractions of the cell-cycle lifetimes. Crescentin recovery may be faster than its other prokaryotic cytoskeletal proteins because crescentin appears to form a stable structure throughout the cell's life cycle while MreB and FtsZ have been shown to condense into a ring structure at the time of cell division, a property that requires both MreB and FtsZ to be more dynamic than Crescentin. Recovery of photobleached crescentin structures in this work (t_1/2_ = 26 min) was faster than previously reported at t_1/2_ = 50 min, where protein synthesis was blocked [2]. This faster recovery rate is possibly due to the synthesis of new crescentin proteins which increase the cytoplasmic pool of unassembled proteins available. Nevertheless, the recovery obtained in both cases are much faster when compared to cytoplamic keratins (∼100 min). Kinetic analysis of the time-dependent FRAP/FLIP intensity profiles in the bleached regions of the cell and *in vitro* assembly results together indicate that the recovery is not limited by the diffusion of unbound crescentin-GFP subunits within the cytoplasmic pool and/or the incorporation of subunits into the already established crescentin structures, but is instead controlled by the slow dissociation of crescentin from those structures.

The possibility of an equilibrium between polymeric structures and subunits has been postulated in IFs [16], [50]–[52]. Following an analogous hypothesis, assembly sites may become available when bleached crescentin-GFP dissociates from polymeric structures and relocates to the cytoplasmic pool. Like other IFs, this subunit exchange may occur throughout the length of the filaments, and not only at filament ends [2], [16], [53], [54]. This model of subunit exchange is more difficult to test for crescentin because of the small size of *C. crescentus* cells compared to cultured mammalian cells.

### Assembly, Ultrastructural, and Mechanical Properties of Crescentin

The ultrastructure of crescentin filaments *in vitro* resembles that of other eukaryotic IFs in standard assembly conditions used for other IF preparations: similar length, diameter, and filament rigidity [34], [37], [55]–[58]. Crescentin's mode of assembly is similar to that of vimentin by increase in solution ionic strength, but different from most other IFs by assembling into filamentous structures upon removal of denaturing buffer (urea) in the absence of nucleotides [55], [59]–[61].

A main function of IFs in higher eukaryotes is to provide cells with mechanical support. Type I and II IF keratins play a key role in the protection of complex epithelia (skin) and simple epithelia (kidney) against mechanical trauma. Type III IF neurofilaments play a key role in shaping the axon of neural cells, while vimentin provides a large fraction of the mechanical rigidity of mesenchymal cells. *In vivo* studies of *C. crescentus* support a similar mechanical function for crescentin. Our rheological studies provide some insight into the mechanical strength of crescentin structures and whether they possess the characteristics to maintain a curved cell shape. Despite the extraordinary diversity of polymer structures formed by IFs [55], [62], they display remarkably similar mechanical properties [30], [34]. Thus far, all tested IFs are stiff, mechanically resilient, more elastic than viscous (i.e. low phase angle), and recover rapidly after shear. These rheological properties stem partially from the high propensity of IFs to form cross-links. Similarly to eukaryotic IFs, crescentin filaments in the presence of divalent cations form extensive cross-links that could promote direct inter-filament interactions.

A rheological signature of IFs in the presence of chemical crosslinks is the relatively low phase angle displayed by crescentin, i.e. crescentin filaments are as solid-like as other eukaryotic IFs. Vimentin, epithelial keratins k5–k14 and k9–k19, crescentin, and nuclear lamin B1 all consistently display a significantly low phase angle of δ∼4–9° (Table 1). The absence of strain hardening is a rheological similarity between bacterial IF crescentin and vimentin, although different from most other eukaryotic IFs. Under shear, some IFs are prevented from bending because of their finite rigidity, and from sliding past one another due to cross-links [34], [35], [57]. These two properties prevent eukaryotic IFs from relaxing stress and, in turn, elasticity builds up in IF networks under shear. This weak inter-filament interaction between crescentin filaments results in a low mechanical resilience of crescentin similar to vimentin. Crescentin softens almost immediately under mechanical stress: the mechanical resilience of crescentin is remarkably low, between 5 and 100 fold lower than that of most other eukaryotic IFs (Table 1). Together these results suggest that the stiffness of crescentin filament suspensions, like that of F-actin suspensions, stems primarily from overlapping or steric interactions between filaments, not from crosslinking interactions between filaments. Divalent ions such as calcium ions appear to increase the width of class III IF vimentin [38]; we do not observe thickening of crescentin filament. The correlation between divalent ions and crescentin function *in vivo* remains to be fully studied, however calcium and magnesium ions are required for the proper growth of *C. crescentus* [63].

Our rheological studies suggest that the observed long-lived mechanical stability of crescentin structures stems not from intrinsically strong inter-filament interactions, but from the crosslinking of crescentin structures to the cell wall by not-yet-identified auxiliary proteins although recent findings [2] show that MreB may mediate the interaction between crescentin and the cell wall. This intereaction may be similar to that observed between their eukaryotic homologs, actin and vimentin [33], [64].

### Crescentin Network Structure Depends on Cationic Valence and Concentration

Polycation-induced lateral filament aggregation is a common feature of cytoskeletal proteins and DNA [65]–[67]. However, rheology and EM suggest that the onset of crescentin filament bundling by Ca^2+^ occurs at concentrations much lower than those required to bundle F-actin. Indeed, actin filament bundling occurs at threshold concentrations of 20 mM Ca^2+^ and 27 mM Mg^2+^ [68], while crescentin filament bundling is observed at concentrations as low as 5 mM Ca^2+^ or Mg^2+^. Linear polyelectrolyte models [67], [69] suggest that this lower filament bundling threshold for crescentin is due to facilitated counterion condensation. Bundling of crescentin is induced by the rapid neutralization of fewer exposed charges on crescentin. The average polymer contour length between charges on DNA and actin is 1.7 Å and 2.5 Å, respectively [68]. If we assume that the cross-section of a crescentin filament has an architecture similar to that of vimentin and that only external monomers would be involved in inter-filament interactions, then we estimate that the average counter length between charges on a crescentin filament is ∼10 Å. This would imply that charges on crescentin indeed require fewer counterions to be neutralized. More precise calculations are clearly needed to fully understand why crescentin has a propensity to bundle in the presence of polycations.

### Potential Physiological Implications

Crescentin's fast filament assembly in vitro leads to the rapid formation of viscoelastic filament networks that may be relevant for its structural role in vivo. Similar to eukaryotic IFs that provide mechanical support in cells either by protecting them against stresses (e.g. keratins in epithelia cells) or by shaping cells (e.g. axon of neural cells), crescentin helps to shape C. Crescentus cells. While crescentin-null *C. crescentus* cells display a rod-shaped phenotype, indicating their participation in shaping the cell, the intrinsic mechanical properties of crescentin suggest that it cannot generate the forces needed to curve the cell wall. Crescentin may directly or indirectly interact with the cell wall through yet to be discovered crescentin-binding proteins that could bundle and crosslink individual filaments, thus increasing their mechanical properties. These binding proteins may be activated by cations, such as calcium, which would neutralize charges on crescentin filaments in a similar manner to the calcium-activated actin-bundling protein fimbrin. In vitro, crescentin rapidly forms stable filament networks that feature mechanical properties similar to IFs while *in vivo*, these structures appear stable and undergo slow remodeling, allowing for little exchange with the pool of unassembled cytoplasmic subunits. Altogether, these properties may be physiologically relevant to enable *C. crescentus* to maintain its crescent shape throughout its life cycle.
